# Nothing lasts forever

**DOI:** 10.3325/cmj.2022.63.584

**Published:** 2022-12

**Authors:** Charles H. Calisher

It has been fun and I am grateful for the opportunities that Matko Marušić, a previous Editor-in-Chief of the *Croatian Medical Journal*, and Svjetlana Kalanj Bognar, the current Editor-in-Chief, have given me to write what I hope have been serious, humorous (sometimes silly), educational, and stimulating essays (“columns”) for the readership of this journal. Marko Kljaković-Gašpić, the journal’s Production Editor also has been extremely supportive and I have enjoyed exchanging views with him. Nonetheless, I have no idea how many people read what I write (“Let’s Get Something Straight”).

I wrote my first column for the *Croatian Medical Journal* in 2006 and over the next 16 years this bi-monthly (every other month, not twice a month; English is a strange and confounding language) journal published 36 of them. I did not write a column for every issue because I took some time off now and again for various reasons. Sometimes it was due to my having run out of ideas or temporarily losing my sense of humor; I do not write columns as easily as summoning a taxi. First, I have an idea. Then I tell my wife what I am thinking of writing. Then she tells me what a stupid idea it is. Then I start over. If she does not tell me the proposed subject and associated opinions are stupid, I take that as approval and get to work. I have taken into account that I am writing for an audience consisting of people whose first language is not English, who may not understand American slang, whose feelings might be hurt by a sentence or phrase I meant to be humorous, or who might become angry at the *Croatian Medical Journal* because of something about which I misspoke. I never worried about the latter because the Editor-in-Chief and the Production Editor would point out the errors and offensiveness of my wordings, so that I could change them.

In reviewing my many visits to Croatia and preparing to write sophisticated columns (an effort soon abandoned), memories of individuals, places, meals, wines, history lessons, personal illnesses, geology, crops, and more came to mind. My principal scientific contact was Dr Jelka Vesenjak Hirjan, then Head of the Department of Virology of Andrija Štampar School of Public Health of Croatia, and her husband, Dr Franjo Hirjan, who was a former Justice of the Supreme Court of Croatia and was influential in our son’s decision to become a lawyer/attorney/*odvjetnik*. Both Jelka and Franjo are now deceased but remain very much alive in my memory. We traveled together in Croatia each fall and I learned a great deal more than I taught. I remain in contact with two of Jelka’s former students, both of whom are accomplished virologists. In addition, the young daughter of a property owner on Brač where we had a study site for our tick collections, spent a year with us as a student in Colorado. My life has been better for having known these wonderful people and we were able to enter our results into the scientific record and to at least temporarily reduce the excess volumes of Dalmatian *crno vino*. That effort ultimately failed, but not because of any lack of trying on our parts.

The American comedian Woody Allen said, “I am not afraid of dying. I just do not want to be there when it happens.” I understand what he meant. I am now 86 years old and have had the usual problems of aging. I have never been 86 before, so I do not know whether upper spine surgery, lower spine surgery, cholecystectomy (followed by pneumothorax), cataract surgery, tenolysis, insertion of a cardiac stent, as well as some less dramatic insults are to be expected but they have been inconvenient. I also have developed difficulties with balance and walking and, although I have consulted with experts and tested for countless problems known to modern medicine, no one has provided me with a reason for this, other than to point out that I am 86, as though I did not know that. One physician looked at my records and declared that I am 100% healthy, except that I cannot walk. I therefore expect to be the healthiest person in the cemetery.

Much worse, my older daughter died two years ago and my older sister died earlier this year, so the long-term view is becoming clearer to me. Therefore, it seems to be time to simplify what remains of my life. I have no intention of taking up golf, a game which does not involve defense, a silly lack. I have decreased the number of manuscripts I am willing to review, withdrawn my memberships in various committees, and surrendered my opportunities to criticize journals and individuals who pretend to understand virus taxonomy.

A book containing 20 of my columns was published by Medicinska Naklada in 2013 and this column is sufficient to complete a second book. I find now to be a good time to quit writing and watch more baseball.

A hearty *hvala vam* to my wife Shelley for her patience and excellent editorial assistance, to the Editors of the *Croatian Medical Journal*, and to whoever has read and enjoyed these columns – my gratitude to all.

“Some cause happiness wherever they go; others whenever they go” – Oscar Wilde

